# Generation of Mature N^**α**^-Terminal Acetylated Thymosin **α**1 by Cleavage of Recombinant Prothymosin **α**

**DOI:** 10.1155/2013/387282

**Published:** 2013-10-28

**Authors:** Bo Liu, Xin Gong, Shaohong Chang, Peng Sun, Jun Wu

**Affiliations:** Department of Microorganism Engineering, Beijing Institute of Biotechnology, 20 Dongdajie Street, Fengtai District, Beijing 100071, China

## Abstract

N^**α**^-terminal acetylation of peptides plays an important biological role but is rarely observed in prokaryotes. N^**α**^-terminal acetylated thymosin **α**1 (T**α**1), a 28-amino-acid peptide, is an immune modifier that has been used in the clinic to treat hepatitis B and C virus (HBV/HCV) infections. We previously documented N^**α**^-terminal acetylation of recombinant prothymosin **α** (ProT**α**) in *E. coli*. Here we present a method for production of N^**α**^-acetylated T**α**1 from recombinant ProT**α**. The recombinant ProT**α** was cleaved by human legumain expressed in *Pichia pastoris* to release T**α**1 *in vitro*. The N^**α**^-acetylated T**α**1 peptide was subsequently purified by reverse phase and cation exchange chromatography. Mass spectrometry indicated that the molecular mass of recombinant N^**α**^-acetylated T**α**1 was 3108.79 in, which is identical to the mass of N^**α**^-acetylated T**α**1 produced by total chemical synthesis. This mass corresponded to the nonacetylated T**α**1 mass with a 42 Da increment. The retention time of recombinant N^**α**^-acetylated T**α**1 and chemosynthetic N^**α**^-acetylated T**α**1 were both 15.4 min in RP-high performance liquid chromatography (HPLC). These data support the use of an *E. coli* expression system for the production of recombinant human N^**α**^-acetylated T**α**1 and also will provide the basis for the preparation of recombinant acetylated peptides in *E. coli*.

## 1. Introduction

Protein acetylation is a common posttranslational modification in eukaryotes but is rarely found in prokaryotes [1–4]. Acetylation has been suggested to play an important biological role in modulating enzymatic activity, protein stability, DNA binding, and peptide-receptor recognition [1]. Charbaut proposed that neutralization of the amino-terminal (N-terminal) positive charge by N^*α*^-acetylation may influence specific interactions with protein partners [3]. For example, nonacetylated rat glycine M-methyltransferase does not exhibit the cooperative behavior of its native counterpart [5]. Acetylation can also affect protein stability. The half-life of nonacetylated-MSH (*α*-melanocyte-stimulating hormone) in rabbit plasma appears to be one-third of that of the acetylated form [6].

Although acetylation is not as common in prokaryotes as in eukaryotes, researchers have identified acetylated forms of recombinant peptides and proteins to be expressed in *E. coli*, examples include interferon *α* and *γ* [7, 8], somatotropin [9], interleukin-10 [10], and chemokine RANTES S24F [11]. However, some of these proteins or peptides, such as interferon *α* and *γ*, are not known to be acetylated in their native forms. Thus, the acetylated forms of these proteins are of little clinical utility.

Thymosin *α*1 (T*α*1) is an immune modifier that has been shown to trigger maturational events in lymphocytes; affect immunoregulatory T-cell function; promote IFN-*α*, IL-2, and IL-3 production by normal human lymphocytes; and increase lymphocyte IL-2 receptor expression [12]. T*α*1 is a 28-amino-acid polypeptide that was originally isolated from thymosin fraction 5. The active peptide, which contains an N^*α*^-terminal acetylated serine residue, is cleaved from prothymosin *α* (ProT*α*) by legumain, a lysosomal asparagine endopeptidase (AEP) in mammals [13]. Given the immunomodulatory properties of N^*α*^-terminal acetylated T*α*1, it is currently being developed for treating several diseases, including hepatitis B and C virus (HBV/HCV) infections, nonsmall cell lung cancer (NSCLC), hepatocellular carcinoma, AIDS, and malignant melanoma [14–18].

To date, N^*α*^-terminal acetylated T*α*1 has been produced by total chemical synthesis for clinical use. However, with 28 residues, it is expensive to produce the peptide by chemical synthesis. As an alternative to synthesis, prokaryotic expression systems are widely used for producing large quantities of recombinant proteins or peptides. When prothymosin *α* (ProT*α*) and its N^*α*^-terminal peptide, T*α*1, were identified 20 years ago, no acetylation was found when they were expressed in *E. coli* [19, 20]. Other groups have expressed recombinant T*α*1 as a fusion protein or in concatemer form in *E. coli *[21–24]. 

We have previously reported that a fraction of recombinant human ProT*α* is N^*α*^-terminal acetylated when expressed in *E. coli* [4]. In this study, we expand on this observation and describe a new method for production and purification of N^*α*^-terminal acetylated T*α*1 from *E. coli.* This method may provide a useful alternative to chemical synthesis for producing acetylated peptides for clinical applications.

## 2. Materials and Methods

### 2.1. Plasmids and Strains

The human ProT*α* expression plasmid pBV220-proT*α* was constructed previously [4]. The yeast *Pichia pastoris* expression plasmid pPIC9, which contains the promoter of the methanol-inducible *P. pastoris *alcohol oxidase 1 (AOX1) gene, the *α*-mating factor prepro secretion signal from *Saccharomyces cerevisiae*, and the HIS4 auxotrophic selection marker, was purchased from Invitrogen (San Diego, CA, USA). The* P. pastoris *GS115 (his4) host strain was from Invitrogen. HepG2 liver carcinoma cell line was purchased from the Institute of Biochemistry and Cell biology (Shanghai, CAS, China).

### 2.2. Production *and* Purification of Recombinant Human Prothymosin *α* in *E. coli*

Recombinant human N^*α*^-acetylated ProT*α* was produced as described previously [4] with some modification. The ProT*α* gene was cloned in plasmid pBV220, with expression under control of the PLPR promoter. Expression of ProT*α* was induced by increasing the culture temperature from 30°C to 42°C for 6 h. After induction, cells were harvested by centrifugation.

The cells were suspended in buffer A (10 mM Tris-HCl, pH 8.0) and incubated at 85°C for 15 min. Acetic acid was added to adjust the pH to 4.0 and to precipitate *E. coli* proteins. Precipitated proteins were spun down, and the supernatant containing ProT*α* was applied on a DEAE Sepharose Fast Flow column (Ø1.6 × 20 cm, GE Healthcare, Piscataway, NJ) equilibrated with buffer A. After washing, the bound protein was eluted with a gradient from 0 to 0.4 M NaCl. Since ProT*α* does not contain any aromatic residues, elution of ProT*α* was monitored by UV absorption at 214 nm. The level of protein and nucleic acid contamination was estimated by UV absorption at 280 nm [25]. The purity of the ProT*α* in eluate fractions was analyzed by 18% SDS-PAGE.

### 2.3. Cloning, Expression and Purification of Recombinant Human Prolegumain in *Pichia pastoris*

Total mRNA was isolated from HepG2 cell line and converted to single-stranded cDNA using MMLV Reverse Transcriptase as described by the manufacturer (TIANGEN BIOTECH Co., Beijing, China). The human legumain gene without signal peptide was amplified by PCR using forward primer legu4 (5′-AGACTCGAGAAAAGAGTTCCTATAGATGATCCTGAAG-3′) and reverse primer legu5 (5′-TATGCGGCCGCTTAGTAGTGACCAAGGCACACGTG-3′); primers were designed based on the ORF of legumain reported previously [26]. Amplification reactions were performed with Pyrobest polymerase (TaKaRa BIOTECH Co., Dalian, China) at 95°C for 2 min, followed by 30 cycles of 94°C for 30 s, 55°C for 40 s, 72°C for 2 min, and a final extension step at 72°C for 10 min. PCR products were cloned into the XhoI and NotI sites (both underlined in primers) of *Pichia pastoris* expression vector pPIC9 to generate the legumain expression vector pPIC9-legumain. The sequence of legumain was verified by sequencing.

Recombinant prolegumain was expressed in *P. pastoris* following the protocol for scale-up expression in shaker flasks as described in the *P. pastoris* expression manual (Invitrogen Life Technologies). The pPIC9-legumain expression vector (10 *μ*g) was digested with SalI. *P. pastoris* GS115 host strain was transfected with the linearized vector by electroporation at 1.5 kV, 129 Ω, and 25 *μ*F in electroporation cuvettes (2 mm gap; BTX). *P. pastoris* colonies growing on MD plates (1.4% yeast nitrogen base, 2% dextrose, 4 × 10^−5^% biotin, and 1.5% agar) were picked for expansion in 50 mL of yeast extract-peptone-dextrose medium (YPD) consisting of 1% yeast extract, 2% peptone, 2% dextrose. Expression of the recombinant legumain was induced by culturing *P. pastoris* in methanol-complex medium containing 1% yeast extract, 2% peptone, 100 mM potassium phosphate buffer, pH 6.0, 1.34% yeast nitrogen base, 4 × 10^−5^% biotin, and 0.5% methanol. Cultures were grown for 72 h, and 0.5% methanol was added every 12 h.

Culture supernatant containing prolegumain was diluted with 5 volumes of buffer C (20 mM sodium acetate, pH 5.5) and applied to SP Fast flow column (Ø1.0 × 16 mm, GE Healthcare, Piscataway, NJ). After washing with buffer C, bound protein was eluted with a step gradient of 25%, 45%, and 100% buffer D (20 mM sodium acetate, 1 M NaCl, and pH 5.5). Eluate fractions were analyzed by 12% SDS-PAGE. Fractions containing recombinant prolegumain were dispensed into aliquots and stored at −70°C until use. 

### 2.4. Autoactivation of Recombinant Human Legumain

To determine the optimum time for autocatalytic activation, 500 *μ*L aliquots of the recombinant prolegumain (0.2 mg/mL) were incubated in 1 mM dithiothreitol (DTT), 1 mM EDTA, and 0.1 M sodium citrate pH 4.0. Samples were incubated for 0 h, 0.25 h, 1 h, and 4 h at 37°C. Digest products were analyzed by SDS-PAGE (12%) and stained with Coomassie Blue.

### 2.5. Cleavage of ProT*α* by Recombinant Human Legumain *In Vitro*

To optimize digestion of ProT*α* by recombinant autoactivated legumain, 250 *μ*L aliquots of recombinant ProT*α* (2 mg/mL) were mixed with 50 *μ*L of legumain in buffer containing 1 mM DTT, 1 mM EDTA, and 0.1 M sodium citrate (pH 4.0). Samples were incubated for 0, 2, 4, 6, 8, and 16 h at 37°C. Digest products were assayed by SDS-PAGE (18%) and stained with Coomassie Blue.

### 2.6. Purification of N^*α*^-Terminal Acetylated T*α*1

Purified ProT*α* (100 mg) was cleaved by autoactivated legumain (1 mg) for 8 h at 37°C. The pH was adjusted to 3.0 by addition of acetic acid. The sample was applied to a PoRos R50 column (Ø1.6 × 20 mm, ABI, Foster City, CA, USA) equilibrated with buffer E (1 M NaCl, 0.1% HCl), and washed with two bed volumes of buffer F (1% ethanol, 0.1% HCl). Protein was eluted with a step gradient of 10%, 20%, and 30% ethanol. Eluate fractions were assayed by RP-HPLC. 

T*α*1-containing fractions were loaded onto an SP High performance column (Ø1.6 × 20 mm, ABI, Foster City, CA, USA) and washed with equilibration buffer M (10 mM NaCl). Bound protein was eluted with 0.2 M NaCl, 0.3 M NaCl, and 0.5 M NaCl. Eluate fractions were assayed by reverse phase-high performance liquid chromatography (HPLC).

### 2.7. RP-HPLC Analysis of N^*α*^-Terminal Acetylated T*α*1

RP-HPLC was performed using the HP HPLC 1090 system. A C18 column (5 *μ*m, Ø4.6 × 250 mm, Johnson Technologies Co. Dalian, China) was equilibrated at a flow rate of 1.0 mL/min in solvent A (0.1% trifluoroacetic acid (TFA) in water). Solvent B was 0.1% TFA in acetonitrile. Samples (25 *μ*L) were injected on the column, and peptides were eluted with a linear solvent gradient (100% A : 0% B to 85% A : 15% B for 5 min; 85% A : 15% B to 75% A : 25% B for 15 min; 75% A : 25% B to 0% A : 100% B for 5 min). Peptide elution was detected by UV-absorption at 214 nm. The column was reequilibrated with solvent A for 20 min between runs.

### 2.8. Mass Spectrometry Characterization and Sequencing of N^*α*^-Terminal Acetylated T*α*1

Purified T*α*1 samples were desalted on a C18 Zip-Tip column (Millipore, Bedford, MA, USA) and eluted in methanol, water, and acetic acid (50 : 50 : 0.5). The molecular mass of N^*α*^-acetylated T*α*1 was determined by nanoelectrospray ionization (nanoESI) quadrupole time-of-fight (Q-TOF MS) using a Q-TOF2 mass spectrometer (Micromass, Manchester, UK). The peptide sequence was determined by tandem mass spectrometry.

## 3. Results and Discussion

### 3.1. Purification of Recombinant Prothymosin *α* Expressed in *E. coli*

**Figure 1:**
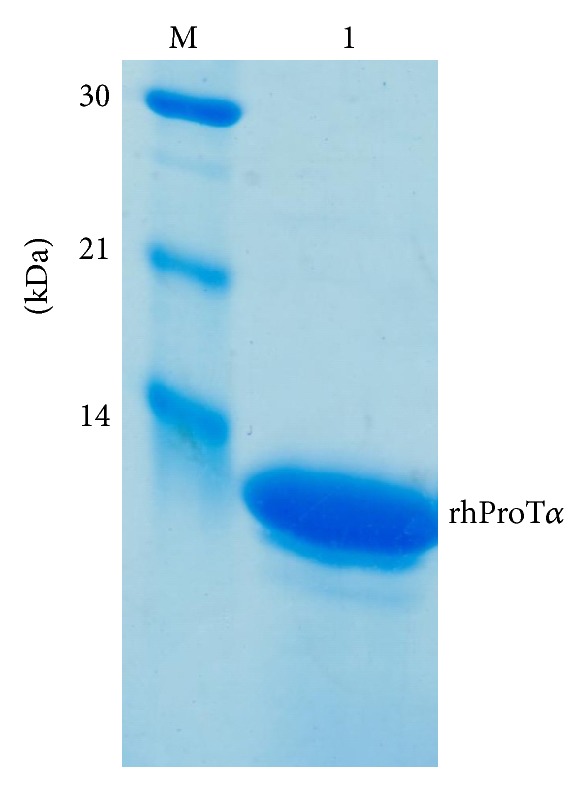
SDS-PAGE of recombinant human prothymosin *α* produced by *E. coli*. The purified recombinant human prothymosin *α* was separated with 18% SDS-PAGE and stained with Coomassie blue G-250. Molecular weight markers were loaded in the same gel (lane M).

Recombinant human prothymosin *α* was overexpressed in *E. coli* [4] and purified by thermal denaturation and anion exchange chromatography. Purified protein appeared as a single homogeneous band with a molecular mass of 12 kDa in 18% SDS-PAGE (Figure 1). 

### 3.2. Preparation of Recombinant Legumain in *Pichia pastoris *and Autoactivation

**Figure 2:**
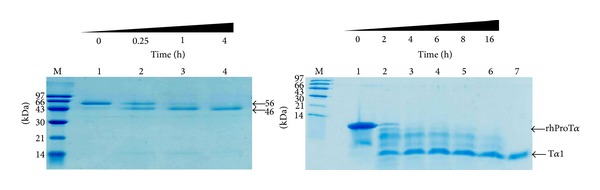
(a) Expression and purification of legumain in *P. pastoris* and its autoactivation. SDS-PAGE of autocatalytic legumain after the prolegumain was incubated in buffer as a function of time (0–4 h) at 37°C. Aliquots of each reaction were analyzed by SDS-PAGE (12%) and stained with Coomassie blue. Each time point is marked on the bottom of its corresponding lane. (b) SDS-PAGE analysis of Prothymosin *α* proteolysis by recombinant legumain *in vitro*. Aliquots of ProT*α* were incubated with recombinant legumain at 37°C in buffer containing 1 mM DTT, 1 mM EDTA, and 0.1 M sodium citrate (pH 4.0). Each reaction mixture was analyzed by SDS-PAGE under the conditions indicated under “Experimental Section.” Each time point is marked on the bottom of its corresponding lane 1–6. Lane 7 is the chemically synthesized N^*α*^-acetylated T*α*1.

We overexpressed human legumain proenzyme, which is reported to cleave ProT*α* at asparaginyl-glycine sites and release peptide products T*α*1 and T*α*11, in *Pichia pastoris*, and purified it by cation exchange chromatography. The molecular mass of recombinant prolegumain was 56 kDa. When incubated at an acidic pH, the recombinant proenzyme was autocleavaged to a 46 kDa form in a time dependent manner (Figure 2(a), lane 2–4).

### 3.3. Prothymosin *α* is Processed to Thymosin *α*1 by Recombinant Legumain *In Vitro*

To process ProT*α* by recombinant legumain *in vitro*, we systematically studied the proteolysis of ProT*α* as a function of pH, temperature, time, and amount of legumain. After incubation with the autoactivated legumain, the band corresponding to ProT*α* decreased in intensity and a band with similar molecular mass of T*α*1 increased in intensity in a time-dependent manner. When 500 *μ*g ProT*α* was incubated with 10 *μ*g legumain, the majority of ProT*α* was cleaved in 2 h, and the cleavage was complete in 6–8 h (Figure 2(b), lane 4-5). Decreasing the amount of legumain also resulted in complete cleavage but required longer incubation times (data not shown).

Legumains have previously been extracted from mammalian and plant cells and tissues [13, 27, 28]. However, these preparations typically have low concentrations and are easily contaminated by other enzymes. This study established a method with which to overexpress the recombinant enzyme in yeast and confirmed its activity in the enzymatic processing of ProT*α*. The yeast expression system is easy and economical to grow on a large scale, and it has been used as an efficient system for production of heterologous proteins [29, 30]. The recombinant prolegumain (56 kDa) incubated at an acidic pH was autocleavaged to a 46 kDa form. This result is consistent with reports that the 56 kDa proenzyme cleaves off the C-terminal 110 residues to form the 46 kDa active enzyme [13].

### 3.4. Purification of the N^*α*^-*Terminal* Acetylated Thymosin *α*1

**Figure 3:**
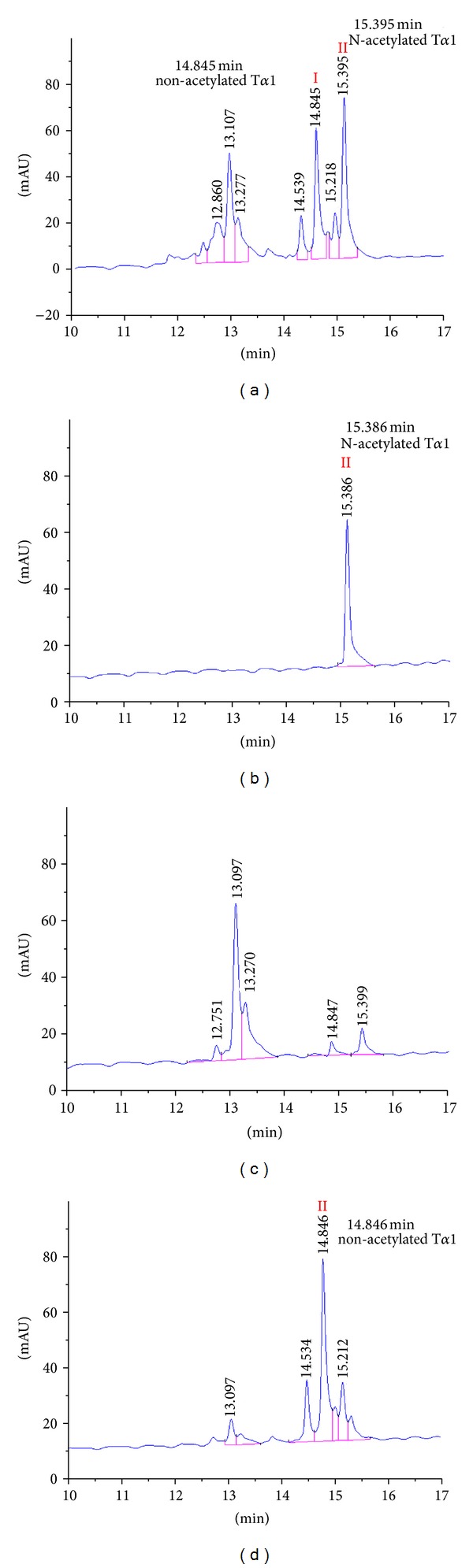
RP-HPLC analysis of N^*α*^-acetylated T*α*1. (a) Sample purified by PoRos R50 column was analyzed by RP-HPLC and eluted as two peaks, I and II corresponding to nonacetylated T*α*1 (14.846 min) and N^*α*^-acetylated T*α*1 (15.396 min). (b–d) RP-HPLC analysis of samples eluted from SP column with (b) 0.2 M NaCl, (c) 0.3 M NaCl, and (d) 0.5 M NaCl. N^*α*^-acetylated T*α*1 was washed out in 0.2 M NaCl fraction (2, peak II), while nonacetylated T*α*1 was washed out in 0.5 M NaCl fraction (4, peak I).

To purify the N^*α*^-acetylated T*α*1 from the ProT*α* cleavage products, the proteolytic products were separated by RP chromatography in PoRos R50 media. Peptide fractions eluted with 10%, 20%, and 30% ethanol were assayed by RP-HPLC, and N^*α*^-acetylated T*α*1 (Figure 3(a), peak II) and nonacetylated T*α*1 (Figure 3(a), peak I) were both identified in 20% ethanol fractions. The acetylated and nonacetylated forms were further separated by cation exchange chromatography. The N^*α*^-acetylated T*α*1 eluted as a single peak (as monitored by UV absorption at 214 nm) in the 0.2 M NaCl elution fraction (Figure 3(b), peak II). Other peptides were found in 0.3 M NaCl and 0.5 M NaCl eluted fractions, including the nonacetylated T*α*1 that eluted with 0.5 M NaCl (Figure 3(d), peak I). Thus, the N^*α*^-acetylated and nonacetylated forms of T*α*1 were completely separated by cation exchange chromatography.

**Figure 4:**
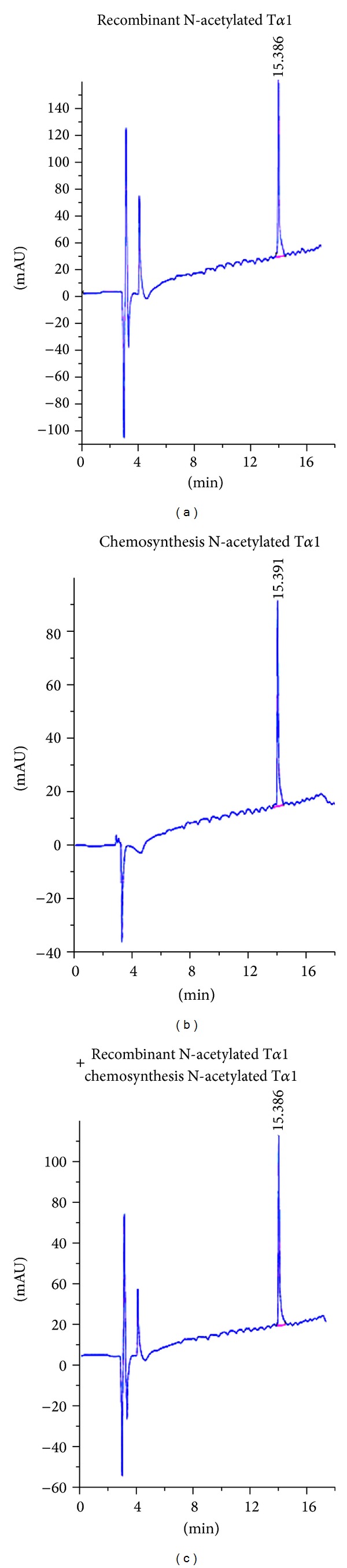
Identification of recombinant N^*α*^-terminal acetylated Thymosin *α*1 by RP-HPLC. Recombinant N^*α*^-acetylated T*α*1 was analyzed by RP-HPLC on a C18 column (5 *μ*m, 4.6 × 250 mm) with a programmed acetonitrile gradient in 0.1% TFA, as detailed under “Materials and Methods.” Chromatograms are shown for (a) recombinant N^*α*^-acetylated T*α*1 (15.386 min), (b) chemically synthesized N^*α*^-terminal acetylated T*α*1 (ZADAXIN, 15.392 min), and (c) mixture of the recombinant T*α*1 and chemically synthesized T*α*1 (15.392 min).

Although ProT*α* and T*α*1 have been overexpressed in *E. coli* previously [19, 22], it was often not determined if the N^*α*^-termianl residue was acetylated. In some cases, this may be due to the difficulty associated with separating N^*α*^-acetylated forms from nonacetylated forms. In this study, we were able to completely separate N^*α*^-acetylated T*α*1 and nonacetylated T*α*1 by HPLC (Figures 3 and 4). The N^*α*^-acetylated T*α*1 was confirmed by MS and MS/MS sequencing.

### 3.5. Identification of the N^*α*^-Terminal *Acetylated* Thymosin *α*1

**Figure 5:**
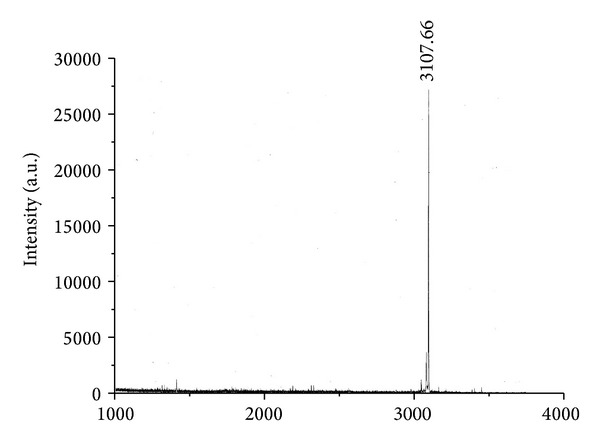
The Q-TOF profile of the recombinant N^*α*^-acetylated T*α*1.

Purified, recombinant N^*α*^-acetylated T*α*1, and chemically synthesized N^*α*^-acetylated T*α*1 were analyzed by RP-HPLC. The elution time of recombinant N^*α*^-acetylated T*α*1 was 15.386 min (Figure 4(a)), which was the same as the N^*α*^-acetylated T*α*1 produced by chemical synthesis (15.392 min, Figure 4(b)). When the recombinant and chemosynthetic forms of N^*α*^-acetylated T*α*1 were mixed and subjected to RP-HPLC, a single peak eluted with a retention time of 15.386 min (Figure 4(c)). The molecular mass of the recombinant N^*α*^-acetylated T*α*1 as determined by Q-TOF mass spectrometry was 3108.79 Da (Figure 5); a mass which is the same as the chemosynthetic peptide and corresponds to the theoretical molecular mass of nonacetylated T*α*1 (3065 Da) plus an additional 42 Da increment for the acetyl group.

The sequence of the recombinant N^*α*^-acetylated T*α*1 was determined by tandem MS. It was found to be “Ac-SDAAVDTSSEITTKDLKEKKEVVEEAEN” (Figure S1a), which is the same as the native and chemosynthetic N^*α*^-acetylated T*α*1. N^*α*^-terminal acetylation was confirmed by a m/z of 130.05 for the N^*α*^-terminal amino acid residue (Figure S1b), which corresponds to an acetylated serine residue.

T*α*1 had been expressed as a fusion protein or in a concatemer form [22, 31, 32]. Zhou et al. reported that 6 × T*α*1 concatemer was successfully expressed in *E. coli* and cleaved by hydroxylamine to release the T*α*1 monomer. However, this version of T*α*1 was not acetylated on the N^*α*^-terminal serine residue [22]. In contrast, this study established an efficient method for preparation of the N^*α*^-acetylated T*α*1 following expression of ProT*α* in *E. coli*. A number of factors make this method easy to scale up for commercial production. Firstly, *E. coli* can be grown to high-density and is capable of producing large quantities of heterologous protein. ZADAXIN, N^*α*^-terminal acetylated T*α*1 produced by total chemical synthesis energizes the immune response, helping patients to battle invasive cancers and secondary infections. It has been tested and proven safe, effective, and easy to tolerate in numerous patient populations, including the elderly, previous treatment failures, and patients with depressed immune systems. In this study, we provide an alternative way to produce N^*α*^-terminal acetylated thymosin *α*1 by cleaving the recombinant protein with legumain, which should be cheaper for large-scale production; as *E. coli* is the most efficient and economic host for recombinant polypeptide production.

In addition, recombinant human legumain is easily expressed as the proenzyme in yeast *P. pastoris* after which it can be autocleaved to yield the active enzyme. Only 2 *μ*g of recombinant legumain is needed to process 400 *μ*g of ProT*α* (data not shown). Finally, the extraction and purification of ProT*α* were based on thermal denaturation [19] and the use of chromatographic media that are chemically stable (can be regenerated repeatedly), commercially available, and relatively inexpensive.

The isolation of N^*α*^-acetylated T*α*1 from recombinant ProT*α* also confirmed our previous findings that ProT*α* is N^*α*^-terminally acetylated in *E. coli* [4]. Acetylation is a common modification in eukaryotic cells, and it plays an important role in protein function and stability. Acetylation, like phosphorylation, can regulate essential cellular processes, such as transcription, nuclear import, microtubule function, and hormonal response [33]. Before this report, a few reports have demonstrated acetylation of proteins expressed in *E. coli*. However, with the exception of N^*α*^-acetylated T*α*1 described here, many of these acetylated proteins are of little clinical utility. N^*α*^-acetylation of proteins is catalyzed by N^*α*^-acetyltransferases (NATs). Three *E. coli* NATs, namely, RimL, RimJ, and RimI, have already been identified through mutant analysis and are responsible for the acetylation of ribosomal proteins L12, S5, and S18, respectively [34, 35]. Fang et al. recently revealed that N^*α*^-acetylation of recombinant fusion proteins of T*α*1 and the ribosomal protein L12 is catalyzed by RimJ in *E. coli* [31]. Johnson et al. reported a applicable method for the expression and purification of functional N-terminally acetylated eukaryotic proteins by coexpressing the fission yeast NatB complex with the target protein in *E. coli* [36]. These results can help reveal the function of acetylation in prokaryotes and find additional mechanisms for producing acetylated proteins in *E. coli*.

## 4. Conclusion

This study established a method for production of recombinant N^*α*^-acetylated T*α*1 in *E. coli*, which is a clinical useful peptide, and was produced by chemosynthesis previously. The product consistent with the chemosynthesis of N^*α*^-acetylated T*α*1, which was confirmed as N^*α*^-acetylated T*α*1 by the elution time in HPLC assay (Figure 3(b)), molecular mass in MS assay (Figure 3(c)), and in MS/MS sequencing (Figure 4). The RP-HPLC showed a single peak in 15.4 min also demonstrated the produced recombinant T*α*1 was a uniform component, with nonacetylated T*α*1 free.

## Supplementary Material

The sequence of recombinant N**α**-terminal acetylated T**α**1 was measured by tandem MS. The sequence of the recombinant N**α**-acetylated T**α**1 was “Ac-SDAAVDTSSEITTKDLKEKKEVVEEAEN”(a), measured by tandem MS of Q-TOF2, which is same as the native and chemosynthesis N**α**-acetylated T**α**1. N**α**-terminal acetylation was confirmed by m/z of 130.05 for the N**α**-terminal amino acid residue, which corresponds to an acetylated serine residue (b).

## Abbreviation

ProT*α*: Prothymosin *α*
Thymosin*α*1: T*α*1
HBV/HBC: hepatitis B and C virus
NSCLC: nonsmall cell lung cancer
Da: Dalton
HPLC: High-performance liquid chromatography
RP: Reverse phase
MS: Mass spectrometry
Q-TOF MS: Quadrupole time-of-fight Mass spectrometry
AEP: Lysosomal asparaginyl endopeptidase
AOX1: Alcohol oxidase 1
NATs: N^*α*^-acetyltransferases
TFA: Trifluoroacetic acid.
