# Supplementary material for: Generation of Mature Nα-Terminal Acetylated Thymosin α1 by Cleavage of Recombinant Prothymosin α
Source: ScientificWorldJournal. 2013 Oct 28;2013:387282. doi: 10.1155/2013/387282 (PMC3830889; doi:10.1155/2013/387282)
Supplement: Supplementary file 1 — The sequence of recombinant Nα-terminal acetylated Tα1 was measured by tandem MS. The sequence of the recombinant Nα-acetylated Tα1 was “Ac-SDAAVDTSSEITTKDLKEKKEVVEEAEN”(a), measured by tandem MS of Q-TOF2, which is same as the native and chemosynthesis Nα-acetylated Tα1. Nα-terminal acetylation was confirmed by m/z of 130.05 for the Nα-terminal amino acid residue, which corresponds to an acetylated serine residue (b). [file 387282.f1.pdf]

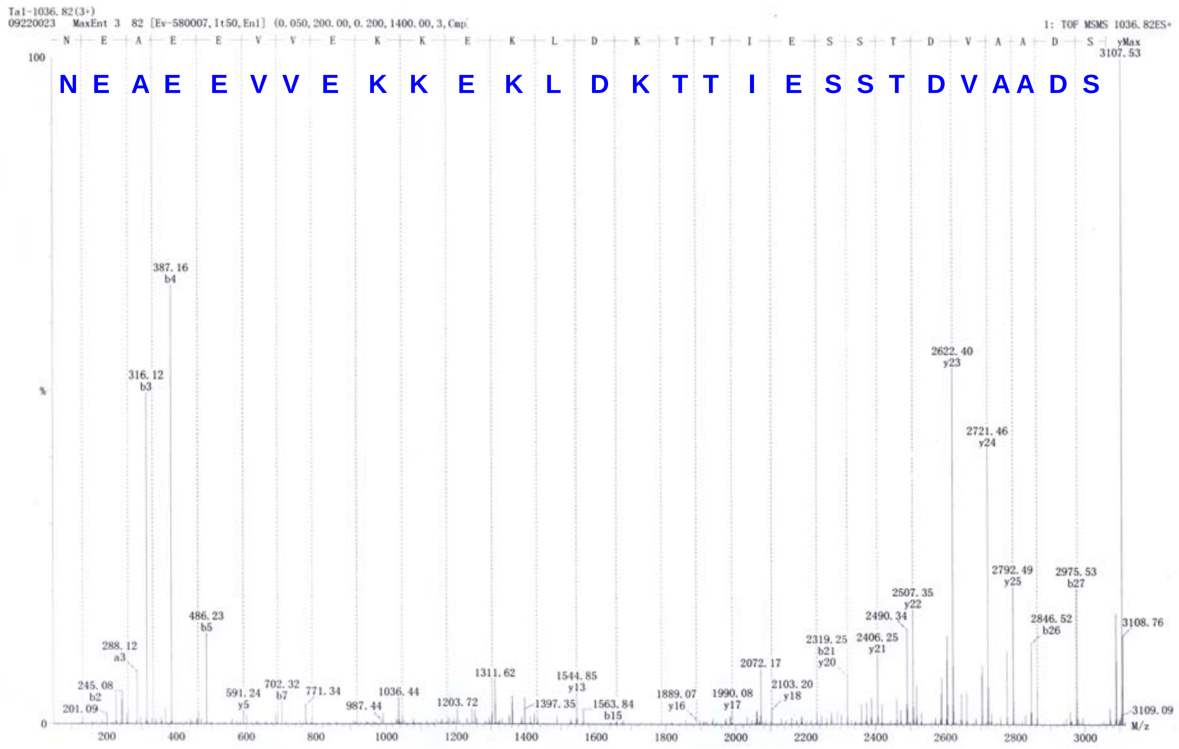

(a)

Observed MW: 3107.4363 Precursor ion charge state: 1
M/z tolerance: 0.30 Intensity threshold: 0 (0.000%)
Modifications: Acetylation (+)

a	102.06	217.08	288.12	359.16	458.23	573.25	674.30	761.33	848.36	977.41
	0.00	0.00	-0.00	-0.01	-0.01	0.01	-0.03	-0.03	-0.07	-0.09
b	130.05	245.08	316.11	387.15	486.22	601.25	702.29	789.33	876.36	1005.40
	0.00	0.00	-0.00	-0.01	-0.01	-0.02	-0.03	-0.04	-0.09	-0.07
	Ser	Asp	Ala	Ala	Val	Asp	Thr	Ser	Ser	Glu
y	3065.50	2978.47	2863.44	2792.41	2721.37	2622.30	2507.27	2406.23	2319.19	2232.16
	----	-0.03	-0.04	-0.08	-0.09	-0.10	-0.08	-0.02	-0.06	-0.10
z	3048.47	2961.44	2846.41	2775.38	2704.34	2605.27	2490.24	2389.20	2302.16	2215.13
	----	-0.14	-0.10	-0.05	-0.16	-0.01	-0.09	-0.10	-0.16	-0.12

a	1090.49	1191.54	1292.59	1420.68	1535.71	1648.79	1776.89	1905.93	2034.02	2162.12
	-0.06	-0.10	-0.07	-0.04	-0.07	-0.04	-0.01	-0.05	0.00	0.13
b	1118.49	1219.53	1320.58	1448.68	1563.70	1676.79	1804.88	1933.92	2062.02	2190.11
	-0.01	-0.03	-0.09	-0.13	-0.13	-0.17	-0.29	-0.02	-0.03	-0.02
	Ile	Thr	Thr	Lys	Asp	Leu	Lys	Glu	Lys	Lys
y	2103.12	1990.04	1888.99	1787.94	1659.84	1544.82	1431.73	1303.64	1174.60	1046.50
	-0.08	-0.05	-0.08	-0.04	-0.14	-0.04	-0.01	-0.09	-0.04	-0.04
z	2086.09	1973.00	1871.96	1770.91	1642.81	1527.79	1414.70	1286.61	1157.57	1029.47
	-0.12	0.26	-0.06	-0.10	-0.15	-0.12	-0.15	-0.03	-0.02	-0.06

a	2291.16	2390.23	2489.30	2618.34	2747.38	2818.42	2947.46	3061.51
	0.17	0.12	0.07	0.03	-0.13	-0.03	-0.04	----
b	2319.16	2418.23	2517.29	2646.34	2775.38	2846.42	2975.46	3089.50
	-0.10	-0.08	-0.07	-0.09	-0.05	-0.10	-0.08	----
	Glu	Val	Val	Glu	Glu	Ala	Glu	Asn
y	918.41	789.36	690.29	591.23	462.18	333.14	262.10	133.06
	-0.02	-0.01	-0.02	-0.01	-0.01	-0.00	-0.00	0.00
z	901.38	772.33	673.26	574.20	445.15	316.11	245.07	116.03
	-0.04	----	-0.01	-0.01	-0.02	-0.01	-0.00	-0.03

(b)
